# Longitudinal association of quality of life and health behaviors with service member readiness

**DOI:** 10.1371/journal.pone.0350971

**Published:** 2026-06-10

**Authors:** Yunnuo Zhu, Sheila F. Castañeda, Isabel G. Jacobson, Crystal L. Lewis, Felicia R. Carey, Scott C. Roesch, Rudolph P. Rull, Millennium Cohort Study Team

**Affiliations:** 1 Deployment Health Research Department, Naval Health Research Center, San Diego, California, United States of America; 2 Leidos, Inc., San Diego, California, United States of America; Japanese Red Cross Medical Center, JAPAN

## Abstract

**Background:**

This study aimed to assess whether self-reported physical and mental health-related quality of life (HRQOL) scores could be useful indicators of military health readiness.

**Methods:**

Survey data and Department of Defense administrative and medical records from 51,589 active duty Millennium Cohort Study participants across four enrollment panels (2001, 2004, 2007, 2011) were examined at baseline and 3- to 5- year follow-up endpoints. Baseline measures included HRQOL measured by the Veterans RAND 12-Item Health Survey (VR-12) mental and physical component summary scores, health behaviors (e.g., smoking, insomnia symptoms), and demographic and military covariates. Readiness outcomes measured at follow-up included health care utilization, separation status, military satisfaction, lost workdays, and non-obese body mass index (BMI). Poisson regression models with a robust error variance were used to examine the prospective relationship between HRQOL and military readiness outcomes.

**Results:**

HRQOL remained a significant predictor of all five readiness outcomes after adjustment for covariates and other health behaviors. Participants with baseline physical HRQOL scores in the highest 15th percentile were more likely to meet readiness metrics (e.g., increased odds of non-obese BMI, fewer annual lost workdays, less annual health care utilization, and a higher likelihood of remaining in service or completing their service term) compared with those in the middle 70th percentile. Scoring in the lowest 15th percentile for baseline physical or mental HRQOL was associated with being less likely to meet readiness metrics 3–5 years later.

**Conclusion:**

Higher physical HRQOL may be a positive indicator of military readiness, while lower physical and mental HRQOL may signal impaired readiness. Assessment of HRQOL using the VR-12 is an easy, patient-centered tool that may help military leaders identify service members needing early interventions and improve readiness and retention.

## Introduction

The effectiveness of the United States (U.S.) military is largely dependent on the readiness of its military force. While readiness is a broad concept, the Department of War (DoW) has used terms such as “operational readiness” and “individual medical readiness” to identify parts of the framework that contribute to overall readiness [1–3]. Operational readiness, or the ability of service members, units, and military systems to properly execute assigned functions and complete mission objectives [1], is critical to optimizing the probability of success in military conflicts [2] while individual medical readiness [3], or the degree to which a service member is physically and mentally fit for duty [4], is a component of operational readiness which sustains unit readiness [1]. For example, meeting physical fitness and weight standards is linked with operational performance [5], while failing to meet these standards [6] is related to military discharges [7,8]. Approximately 75% of active duty personnel have a body mass index (BMI) in the overweight (25.0–29.9 kg/m^2^) or obese ranges (≥30 kg/m^2^) [9]. The inability to meet physical requirements due to obesity affects both recruitment and retention. Service members with high BMI have more health care visits and are more likely to separate from service earlier than service members with a healthy BMI [10,11]. This poses a threat to health readiness and costs the DoW over a billion dollars per year [11].

Studies suggest that mental health conditions (e.g., posttraumatic stress disorder [PTSD], depression) deter health readiness [12,13] and are associated with lost workdays [14] and early attrition from the military. Other markers of readiness include job satisfaction and job retention [15,16], which contribute to maintenance of force strength and reduce unnecessary costs associated with early attrition [1,17]. Time away from the job due to illness and injury also affects readiness. Chronic health conditions and non-fatal work-related injuries lead to lost workdays and diminished productivity that incur significant costs to the DoW and Military Health System (MHS) [18,19]. While preventive care (e.g., routine wellness exams, vaccinations) ensures that service members are medically ready to serve, seeking health care beyond preventive care may be indicative of conditions or injuries that negatively impact readiness and increase costs to the MHS [20,21].

Readiness depends on the adjustment and satisfaction of service members, which are heavily influenced by well-being, quality of life [22,23] and health behaviors. Sleep health has gained attention over the last several years within the DoW due to its impact on readiness [24–26] and associated health outcomes [27,28]. Sleep disturbances such as insomnia affect physical and mental performance of service members [29]. Fatigue and reporting less than the recommended hours of sleep has been associated with an increased risk of accidents or mistakes during combat deployments [30]. In addition, tobacco use and high alcohol consumption increase the risk of chronic diseases and associated health care utilization, which cost the DoW $989 million per year for related medical care [31]. Active duty heavy drinkers reported greater loss of productivity compared with moderate drinkers [32], and smoking has been shown to be an important indicator of physical and mental health [33], demonstrating that substance use may impede readiness. These unhealthy behaviors impose costs and productivity loss that reduce readiness while health-promoting lifestyles produce savings for the MHS [20,21].

The identification of health factors associated with readiness within military populations can inform strategies to improve health, well-being, and readiness among service members and their families. However, current screening tools in use do not fully encompass behavioral, psychosocial, and health-related quality of life (HRQOL) factors that may be crucial to promoting service member readiness. HRQOL is the emotional and physical capacity of an individual to function in their daily lives [34]. While there is evidence that certain behaviors (e.g., sleep or substance use) impact readiness and retention, little is known about how such behaviors in conjunction with HRQOL predict readiness.

The DoW characterizes readiness as the military’s ability to create, deploy, and sustain its warfighters and warfighting units to meet mission demands [1]. The definition of readiness has changed as the abilities and demands of the military services have evolved, but individual service member readiness remains foundational. This study focuses on personnel readiness using key ideas from the Total Force Fitness framework which identifies the need to evaluate mind and body through eight domains including physical, psychological, nutritional, social, spiritual, environmental, financial, and medical needs among individuals to maintain a resilient and ready military force [35,36]. The measures of readiness selected for this study, military service satisfaction, separation status, lost workdays, health care utilization, and BMI, reflect these domains through the actions and experiences of individual service members. Military job satisfaction is used as a measure of readiness in this study as it addresses service member’s attitude about military service and is directly associated with service retention [37,38]. Early separation due to medical, social, or psychological reasons such as PTSD or depression, and the resulting inability to retain trained service members costs the military time and money [11]. These measures highlight the importance of consistent staffing and sustainability. Lost workdays due to injury, physical or psychological illness, non-preventive health care utilization, and having an obese BMI are physical, psychological, and medical measures of readiness. These outcomes reflect not just immediate physical, psychological, and medical health demands but also represent training readiness through loss of training knowledge or skills due to inability to attend or complete training tasks through absence or physical impediment.

While readiness was once seen as solely a physical measure, addressing these broader measures allows for a more complete picture of what comprises readiness among military personnel [36]. By measuring how HRQOL is associated with these varied measures of readiness outcomes, in conjunction with baseline health factors, the study aimed to assess the potential utility of HRQOL as a meaningful clinical screening tool to assess service member readiness.

## Methods

### Study population

**Fig 1:**
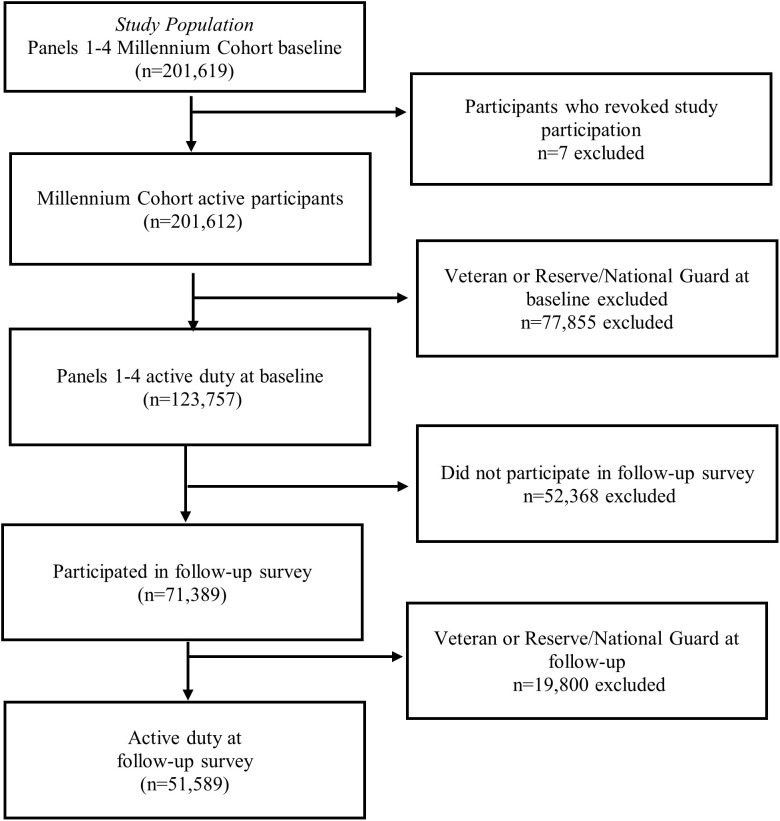
Participant flow chart.

The US Millennium Cohort Study is the largest and longest-running longitudinal study of military personnel and is sponsored by the DoW and the Department of Veterans Affairs. The goal of the study is to evaluate the impact of military service, including deployments and other occupational experiences, on the long-term health of service members and veterans. Millennium Cohort Study participants comprise all branches of service, as well as active duty, Reserve, and National Guard components. To date, more than 260,000 service members have enrolled across five panels, the first of which was enrolled in 2001, with subsequent panels enrolled in 2004, 2007, 2011, and 2020. Participants provided written informed consent at enrollment, are surveyed approximately every 3–5 years after their initial enrollment, and are followed throughout the life cycle of their military career and after separation from service [38,39]. For the current study, the eligible sample was drawn from the first four panels (n = 201,619) enrolled from 01/07/2001 to 30/06/2003, 01/06/2004 to 14/02/2006, 05/06/2007 to 31/12/2008, and 07/06/2011 to 04/04/2013 after removing participants who withdrew from the study and revoked permission for continued use of their data (n = 201,612) (Fig 1). The sample included those who were active duty at baseline (n = 123,757), remained on active duty through their first follow-up survey approximately 3–5 years later (n = 71,389), and completed both the baseline survey and first subsequent follow-up survey (n = 51,589). Active duty participants who completed their baseline survey and who accessed the MHS at least once during the 3–5 year follow-up period were included in health care utilization models (n = 50,853). All participants provided informed voluntary consent and the study was approved by the Naval Health Research Center Institution Review Board (protocol number NHRC.2000.0007).

### Measures

Study data were obtained on 13/10/2020 from participants’ baseline survey (2001–2013 based on enrollment panel) and self-reported outcomes obtained from their first follow-up survey (2004–2016) captured 3–5 years later. Administrative data from Defense Manpower Data Center (DMDC) and electronic health records from the MHS Data Repository (MDR) were used to capture deployment and separation status, and health care utilization using Current Procedural Terminology codes. DMDC administrative data was obtained on 11/05/2021 and MDR data was accessed on 07/04/2021; both were deidentified before analyses were conducted.

**Readiness outcomes.** Readiness outcomes were assessed at the follow-up survey unless otherwise noted.

**Military service satisfaction** was measured by one self-report item asking participants about their overall feelings regarding their military service, with five response options ranging from negative to positive. This measure was not available on follow-up surveys for panel 1 participants (2004–2006); only participants in panels 2–4 (2007–2016) are included in this analysis (N = 26,855). **Separation status** was identified using military separation codes from throughout each service member’s service history. Service members who left before the end of their term of service for reasons such as misconduct, disability, or failure to meet fitness standards were identified as early separators [29]. Participants with routine separations, retirements, or those that were still active duty as of September 30, 2023 were considered active service or routine separators. **BMI** was calculated from self-reported height and weight, and service members with a BMI ≥ 30 kg/m^2^ were considered as having obesity. **Lost workdays** were measured by the self-reported number of days in the past 3 years of missed work due to illness or injury. **Health care utilization** was measured as the number of days per year of cumulative MDR inpatient and outpatient visits between baseline survey and follow-up survey dates, excluding preventive outpatient visits such as annual physicals, occupation-related Periodic Health Assessments, or pregnancy-related checkups. Only participants who utilized the MHS at least once between enrollment and follow-up survey dates were included in models examining health care utilization. Service members who indicated either positive military service satisfaction, did not have an early separation code, had a non-obese BMI (<30 kg/m^2^), missed less than 6 days of work due to injury or illness in the past 3 years, or utilized less than 6 days of non-preventive health care per year were considered to be ready.

**Baseline health factors.** All predictors were assessed at the time of baseline enrollment unless noted otherwise.

**HRQOL** was assessed using the Veterans RAND 36-Item Health Survey (SF-36V), a standardized and widely used brief assessment that asks about the self-appraised effect of somatic (“physical”) and emotional (“mental”) health problems on basic day-to-day functioning [40–42]. The Veterans RAND 12-Item Health Survey (VR-12) was developed from the SF-36V, which was developed and modified from the original RAND version of the 36-item Health Survey version 1.0 (Medical Outcomes Study SF-36). All SF-36V survey responses were recoded to VR-12 summary scores using a validated scoring algorithm [42–44]. The 12 items are weighted to yield the **mental component summary (MCS)** score and **physical component summary (PCS)** score. MCS (range: −2.6 to 71.7) and PCS (range: 7.6 to 72.2) scores have normative values with a mean of 50 and standard deviation of 10 to compare with other US populations, with higher scores reflecting a more favorable health status [44–46]. MCS and PCS scores were categorized into three groups (highest 15th percentile, middle 70th percentile, and lowest 15th percentile) as used in prior Millennium Cohort Study research [47–52] for greater utility and applicability for use as a screening tool for readiness.

**Probable depression** was measured using the 8-item patient Health Questionnaire (PHQ-8) [53]; participants screened positive for probable depression if responses to questions met the threshold based on Diagnostic and Statistical Manual of Mental Disorders, 5th Edition (DSM-5) criteria, including endorsing “more than half the days” or “nearly every day” for five or more of the eight depressive symptoms, of which one item was depressed mood or anhedonia [54].

**Probable posttraumatic stress disorder** (PTSD) was screened for using the 20-item PTSD Checklist for DSM-5 (PCL-5) and scored based on criteria in the DSM-5 [54,55]. Endorsement of at least one intrusion item, two or more hyperarousal symptoms, and at least three avoidance symptoms were necessary to screen positive for probable PTSD.

**Deployment and combat exposure** between baseline and follow-up survey were identified using deployment dates from DMDC records while combat exposure was assessed using a 13-item combat experience measure.

**Insomnia symptoms** were measured using questions from two validated instruments, the Primary Care Evaluation of Mental Disorders Patient Health Questionnaire for anxiety screening and the PTSD Checklist–Civilian Version [53,54]. Both items ask participants about whether they have had trouble falling asleep or staying asleep. Responses were combined to determine whether individuals had no insomnia symptoms on both instruments [29], or insomnia symptoms on one or both screening instruments.

**Sleep duration** was captured by a single item asking participants the number of hours slept in an average 24-hour period. Service members who reported sleeping 7–9 hours on average per night were classified as having recommended levels of sleep, while those sleeping less than 7 or greater than 9 hours were classified as sleeping below or above recommended levels, respectively [56].

**Smoking status** was assessed as never, former, or current smoking. Smoking status was based on lifetime cigarette use and quit attempts, classified as ever smokers if participants reported that they had smoked at least 100 cigarettes (five packs) in their lifetime and never smokers if they had not; ever smokers were further classified as former smokers if they reported having quit successfully or not having smoked in the past year [57].

**Binge drinking** was defined as drinking more than recommended sex-specific limits based on self-reported consumption of four or more drinks per occasion for women and five or more drinks per occasion for men [58].

**Baseline BMI** was calculated from self-reported height and weight using the same process as follow-up BMI. Service members with a BMI ≥ 30 kg/m^2^ at baseline were considered as having obesity.

**Self-reported provider diagnoses** were defined as self-reporting whether a doctor or health professional ever told participants they had any of the five most common health diagnoses in this study population, which included sinusitis, migraine headache, hypertension, depression, or bladder infection.

**Number of stressful life events** assessed whether participants had ever reported one or more stressful life events, including suffering a disabling illness or injury, divorce or marital separation, major financial problems (e.g., bankruptcy), a family member or loved one becoming severely ill or dying, a violent assault, forced sexual relations or assault, and sexual harassment.

### Covariates

Demographics, including sex, race, ethnicity, and birth date were obtained from the DMDC database at study enrollment. These demographic characteristics are self-reported by individuals when they enroll into military service. Age at baseline survey completion was calculated using participants’ survey completion dates and birth dates. Marital status and education were ascertained from the survey and backfilled using administrative data from DMDC if survey data were missing. Pay grade (enlisted vs. officer) and service branch were obtained from DMDC at study enrollment. Enrollment panel (1–4) was also included as a covariate.

### Statistical analyses

Descriptive frequencies of baseline health factors and covariates were reported for the overall study sample. Based on a variance inflation factor threshold of ≥4, no multicollinearity was observed in any of the study models. Poisson regression models with a robust error variance were used to assess the association between HRQOL and readiness outcomes, adjusting for baseline health factors, demographic and military characteristics, and study enrollment panel. Prevalence ratios were reported due to the high prevalence (>10%) of all selected readiness outcome measures, which may lead to overestimation of the association between predictor and outcome variables with odds ratios in a logistic regression [59]. Risk ratios were not examined, as study measures are only measured at each data collection time point and represent point prevalence, rather than incident cases.

The proportion of missing data among covariates in the analytic sample ranged from 0.01% to 4.44%. Only complete cases were used in all Poisson regression models. All *p*-values were two-sided, and values < .05 were considered statistically significant. Sensitivity analyses were performed using continuous MCS and PCS scores. All statistical analyses were conducted using SAS software version 9.4 (SAS Institute, Inc., Cary, North Carolina, USA).

## Results

### Sample characteristics

Table 1 presents the sample characteristics for active duty service members along with the prevalence of HRQOL, health behaviors, and readiness outcomes. Among the 51,589 participants, scores ranged from 6.7 to 79.4 for physical HRQOL and −1.8 to 76.2 for mental HRQOL, with 10.2% and 14.8% of the sample scoring in the highest 15th percentile for mental and physical HRQOL, respectively. At baseline, most participants were under the age of 35 years (78.2%), married (62.8%), serving in the Air Force (36.1%) or Army (35.4%), and in enlisted ranks (73.2%). More than half of service members reported no insomnia symptoms (62.6%), while 45.2% reported 7–9 hours of sleep; 45.5% reported no binge drinking; and 61.6% never smoked. Service members most frequently reported only one stressful life event in the past three years (43.0%) and no commonly occurring health diagnoses (73.2%).

**Table 1:** Service Member Characteristics, Health Factors, and Readiness Outcomes (N = 51,589).

Study Population Characteristics	n	%
**Sociodemographic (at baseline)**		
**Time to follow-up, years (mean, SD)**	3.05	0.83
**Age group, years**		
17-24	13,861	26.9
25-34	26,465	51.3
35+	11,263	21.8
**Sex**		
Male	37,807	73.3
Female	13,782	26.7
**Race and ethnicity**		
American Indian or Alaska Native, non-Hispanic	710	1.4
Asian or Pacific Islander, non-Hispanic	2,737	5.3
Black, non-Hispanic	6,447	12.5
Hispanic/Latino	4,163	8.1
Multiracial	672	1.3
White, non-Hispanic	36,855	71.5
Missing	5	0.01
**Marital status**		
Never married	14,521	28.2
Married	32,378	62.8
Divorced/widowed/separated	4,685	9.1
Missing	5	0.01
**Service branch**		
Air Force	18,624	36.1
Army	18,249	35.4
Coast Guard	1,398	2.7
Marine Corps	3,621	7.0
Navy	9,697	18.8
**Pay grade**		
Enlisted	37,785	73.2
Officer	13,804	26.8
**Panel (baseline survey years)**		
1 (2001-2003)	22,339	43.3
2 (2004-2006)	7,532	14.6
3 (2007-2008)	10,160	19.7
4 (2011-2013)	11,558	22.4
**Health factors (baseline)**		
**Probable PTSD**		
No	48,641	94.3
Yes	1,924	3.7
Missing	1,024	2.0
**Probable Depression**		
No	49,856	96.6
Yes	1,230	2.4
Missing	503	1.0
**Deployment**		
Did not deploy	18,579	36.0
Deployed, no combat	12,851	24.9
Deployed, with combat	20,054	38.9
Missing	105	0.2
**Body mass index, kg/m** ^**2**^		
<30	47,166	91.4
≥30	3762	7.3
Missing	661	1.3
**Insomnia symptoms**		
None	32,297	62.6
Any symptoms	18,980	36.8
Missing	312	0.6
**Sleep duration (hours)**		
7-9	23,324	45.2
<7 or>9	28,265	54.8
**Binge drinking**		
Yes	26,923	52.2
No	23,324	45.5
Missing	1,197	2.3
**Smoking**		
Never	31,778	61.6
Former	10,793	20.9
Current	8,497	16.5
Missing	521	1.0
**Stressful life events (range: 0–7)**		
0	14,442	28.0
1	22,173	43.0
2+	14,033	27.2
Missing	941	1.8
**Number of diagnoses (range: 0–5)**		
0	37,749	73.2
1+	13,086	25.4
Missing	754	1.5
**Mental HRQOL (range: −2.6 to 71.7)**		
Highest 15% (>58.31)	7,751	15.0
Middle 70% (42.98–58.31)	35,588	69.0
Lowest 15% (<42.98)	7,641	14.9
Missing	609	0.8
**Physical HRQOL (range: 7.6 to 72.2)**		
Highest 15% (>57.91)	7,645	14.8
Middle 70% (48.95–57.91)	35,860	69.5
Lowest 15% (<48.95)	7,678	14.9
Missing	406	0.8
**Readiness outcomes (follow-up)**		
**Separation status**		
Early separation	5,311	10.3
Routine separation/active duty	46,065	89.3
Missing	213	0.4
**Non-preventive health care utilization (inpatient/outpatient), days/year**		
0-5	34,814	67.5
6+	16,019	31.1
Missing	746	1.4
Military satisfaction*		
Positive	19,453	37.7
Neutral/ Negative	7,402	14.3
Missing	24,734	47.9
**Lost workdays in past 3 years**		
0-5	36,233	70.2
6+	13,315	25.8
Missing	2,041	4.0
**Body mass index, kg/m** ^**2**^		
<30	42,848	83.1
≥30	6,187	12.0
Missing	2,554	5.0

PTSD: posttraumatic stress disorder; HRQOL: health related quality of life

*Military satisfaction data were only available for panels 2–4.

Most of the sample met readiness metrics at follow-up, with an average time between baseline and follow-up surveys of 3.05 years (SD = 0.83). Specifically, most participants were either still serving or separated for routine reasons (89.3%) and 45.5% missed one work week or less (0–5 days) on average per year due to illness or injury. Most service members (67.5%) had 0–5 days of inpatient or outpatient care in the MHS in the year following their baseline survey. The majority had a non-obese BMI (83.1%), and among those with data on military service satisfaction, 72.4% reported positive feelings about their service (Table 1).

### HRQOL and health factors as predictors of readiness

Table 2 lists results from the multivariable regression models for the five readiness outcomes.

**Table 2:** Adjusted Prevalence Ratios of Health Behaviors and Health-Related Quality of Life (HRQOL) on Readiness Outcomes.

	Non-Obese BMI (Ref: > 30 kg/m^2^)		Five or Fewer Annual Lost Workdays (Ref: ≥ 6 days/ year)		Five or Fewer Days of Annual Non-Preventive Health Care Utilization (Ref: ≥ 6 days of visits/year)		Routine Separation/ Active Duty (Ref: Early separation)		Military Satisfaction (Ref: Neutral/ Negative satisfaction)	
	N = 46,021		N = 46,537		N = 47,700		N = 48,206		N = 24,900	
	APR	95% CI	APR	95% CI	APR	95% CI	APR	95% CI	APR	95% CI
Mental HRQOL										
Highest 15th percentile	**1.07**	**1.01-1.13**	1.03	0.98-1.07	0.96	0.91-1.00	**1.10**	**1.01-1.20**	**1.27**	**1.17-1.38**
Middle 70th percentile	Ref.		Ref.		Ref.		Ref.		Ref.	
Lowest 15th percentile	**0.94**	**0.88-0.99**	**0.88**	**0.85-0.92**	**0.90**	**0.86-0.95**	**0.90**	**0.84-0.97**	**0.76**	**0.72-0.80**
Physical HRQOL										
Highest 15th percentile	**1.24**	**1.15-1.34**	**1.17**	**1.11-1.23**	**1.12**	**1.07-1.19**	**1.11**	**1.01-1.20**	1.05	0.99-1.11
Middle 70th percentile	Ref.		Ref.		Ref.		Ref.		Ref.	
Lowest 15th percentile	**0.93**	**0.89-0.98**	**0.65**	**0.63-0.67**	**0.68**	**0.65-0.71**	**0.74**	**0.70-0.79**	**0.80**	**0.76-0.84**
Probable PTSD										
No	1.11	1.00-1.23	1.02	0.95-1.09	1.04	0.96-1.12	**1.17**	**1.05-1.31**	1.04	0.96-1.13
Yes	Ref.		Ref.		Ref.		Ref.		Ref.	
Probable Depression										
No	1.12	0.99-1.27	0.97	0.90-1.05	1.01	0.92-1.11	**1.18**	**1.05-1.31**	**1.18**	**1.08-1.29**
Yes	Ref.		Ref.		Ref.		Ref.		Ref.	
Deployment										
No deployment	Ref.		Ref.		Ref.		Ref.		Ref.	
Deployed, no combat	1.01	0.95-1.07	**1.22**	**1.16-1.27**	**1.32**	**1.26-1.38**	1.05	0.97-1.14	**1.11**	**1.04-1.18**
Deployed, combat	0.95	0.90-1.00	0.98	0.94-1.01	**1.24**	**1.19-1.29**	**0.94**	**0.88-1.00**	**1.10**	**1.04-1.16**
Non-Obese BMI at Baseline	**9.37**	**8.98-9.77**								
No	**9.37**	**8.98-9.77**	**1.14**	**1.09-1.20**	**1.22**	**1.15-1.30**	**1.48**	**1.36-1.62**		1.05 (0.97-1.13)
Yes	Ref.		Ref.		Ref.		Ref.		Ref.	
Insomnia Symptoms										
None	**1.07**	**1.02-1.12**	**1.17**	**1.13-1.21**	**1.17**	**1.13-1.22**	**1.09**	**1.03-1.16**	**1.14**	**1.09-1.19**
Any symptoms	Ref.		Ref.		Ref.		Ref.		Ref.	
Sleep duration (hours)										
7-9	**1.09**	**1.05-1.14**	**1.07**	**1.04-1.11**	**1.06**	**1.02-1.10**	1.05	1.00-1.12	**1.15**	**1.10-1.21**
<7/> 9	Ref.		Ref.		Ref.		Ref.		Ref.	
Binge Drinking										
No	**1.07**	**1.03-1.12**	1.03	1.00-1.06	**0.93**	**0.90-0.97**	0.99	0.93-1.04	0.98	0.94-1.02
Yes	Ref.		Ref.		Ref.		Ref.		Ref.	
Smoking										
Never	**0.87**	**0.82-0.93**	**1.05**	**1.01-1.09**	1.01	0.97-1.06	**1.20**	**1.12-1.28**	1.05	0.99-1.10
Former	**0.89**	**0.83-0.95**	0.99	0.95-1.04	0.95	0.91-1.00	**1.12**	**1.04-1.20**	**1.07**	**1.01-1.13**
Current	Ref.		Ref.		Ref.		Ref.		Ref.	
Stressful Life Events										
0	1.06	1.00-1.12	**1.29**	**1.24-1.35**	**1.21**	**1.15-1.27**	**1.12**	**1.04-1.20**	1.06	1.00-1.12
1	0.99	0.95-1.05	**1.18**	**1.14-1.22**	**1.14**	**1.09-1.18**	**1.13**	**1.06-1.20**	**1.09**	**1.04-1.15**
2+	Ref.		Ref.		Ref.		Ref.		Ref.	
Number of top 5 diagnoses*										
0	**1.08**	**1.03-1.13**	**1.28**	**1.24-1.32**	**1.30**	**1.26-1.35**	**1.14**	**1.07-1.21**	**1.10**	**1.05-1.15**
1+	Ref.		Ref.		Ref.		Ref.		Ref.	

BMI: body mass index; APR: adjusted prevalence ratio; CI: confidence interval.

*Top 5 diagnoses include sinusitis, migraine headache, hypertension, depression, bladder infection.

Model adjusted for demographics, education, baseline BMI, military factors, and survey panel. Bolded values indicate a p value less than 0.05.

**BMI.** Non-obese BMI at baseline was the strongest predictor of non-obese BMI at follow-up (Adjusted Prevalence Ratio [APR] = 9.37, 95% Confidence Interval [CI]: 8.98–9.77). Participants who scored in the top 15th percentile for mental or physical HRQOL were, respectively, 7% and 24% more likely to have a non-obese BMI compared with participants scoring in the middle 70^th^ percentile (95% CI: 1.01–1.13 and 1.15–1.34, respectively). Those who scored in the bottom 15th percentile for mental or physical HRQOL were more likely to have an obese BMI compared with those in the middle 70th percentile. Health-promoting behaviors (getting the recommended hours of sleep, not binge drinking, and not smoking) were significantly associated with having a non-obese BMI. Probable depression, PTSD, and stressful life events were not significantly associated with BMI in this population.

**Lost workdays in past 3 years.** Participants who scored in the top 15th percentile for physical HRQOL were 18% more likely to miss five or fewer workdays compared with participants scoring in the middle 70th percentile (95% CI: 1.12–1.24). Those scoring in the bottom 15th percentile for mental or physical HRQOL were less likely to miss five or fewer workdays compared with those in the middle 70th percentile. Having no insomnia symptoms, sleeping for 7–9 hours a night, and never having smoked were significantly associated with fewer lost workdays. Reporting 1 or fewer stressful life events compared with 2 or more were also associated with fewer lost workdays. Probable PTSD and depression were not associated with lost workdays in this population.

**Annual health care utilization.** Participants in the top 15th percentile for physical HRQOL were 14% more likely to have five or fewer days of non-preventive health care utilization compared with participants scoring in the middle 70th percentile (95% CI: 1.018–1.20). Those who scored in the bottom 15th percentile for mental or physical HRQOL were less likely to have five or fewer days of non-preventive health care visits compared with those in the middle 70th percentile. Most health-promoting behaviors or fewer stressful life events were significantly associated with fewer health care visits, except for smoking (never smoked: APR = 1.01, 95% CI: 0.96–1.06.; formerly smoked: APR = 0.95, 95% CI: 0.90–1.00). Probable PTSD and depression were not associated with fewer health care visits in this population. Participants who deployed, regardless of combat exposure, were more likely to have five or fewer days of non-preventive health care utilization compared with participants who did not deploy.

**Separation status.** Participants who scored in the top 15th percentile for mental and physical HRQOL were, respectively, 10% and 12% more likely to separate for routine reasons or remain on active service than those who scored in the middle 70th percentile (APR = 1.10, 95% CI: 1.01, 1.19, APR = 1.12, 95% CI: 1.03, 1.21, respectively). In contrast, participants scoring in the lowest 15th percentile for physical (APR = 0.73, 95% CI: 0.68, 0.78) and mental (APR = 0.90, 95% CI: 0.84, 0.97) HRQOL were less likely to separate for routine reasons or remain in service than those scoring in the middle 70th percentile. Insomnia, smoking, stressful life events were statistically significantly associated with separation status, while sleep duration and binge drinking were not. Participants who did not screen positive for probable PTSD or depression were more likely to remain in the military or separate for routine reasons.

**Military satisfaction.** Service members scoring in the top 15th percentile for mental HRQOL were 28% more likely to have positive (versus neutral or negative) feelings about their military service compared with those who scored in the middle 70th percentile (95% CI: 1.18–1.39), while there was no significant association between scoring in the highest 15th percentile for physical HRQOL and positive feelings about military service (Table 2). Those scoring in the lowest 15th percentile for mental or physical HRQOL were less likely to have positive feelings compared with those scoring in the middle 70th percentile. Participants who screened negative for probable depression were more likely to report positive satisfaction with their military service whereas there was no association between military satisfaction and probable PTSD. Those who deployed, regardless of combat exposure, were more likely to report positive military satisfaction compared with those who did not deploy. In terms of other health factors, participants with no insomnia or who slept the recommended 7–9 hours a night were more likely to report positive military satisfaction than those with insomnia symptoms or who slept more or less than the recommended 7–9 hours. Binge drinking was not associated with military satisfaction. Those who formerly smoked were more likely to report positive military satisfaction compared with those who currently smoke while there was no association between never smoking and military satisfaction. Having one stressful life event compared with 2 or more was associated with positive military satisfaction while reporting no stressful life events had no association with military satisfaction.

Sensitivity analyses were performed for all models using continuous MCS and PCS scores (results not shown) with results displaying significant directionality consistent with models using categorical percentile cut-offs. Prevalence ratios for MCS and PCS showed a 1–2% increase in prevalence of readiness outcomes for each 1 unit increase in MCS score, and a 1–3% increase in prevalence of readiness outcomes for each 1 unit increase in PCS score. Continuous MCS score had the highest increase in prevalence associated with military satisfaction (APR = 1.02, 95% CI: 1.02, 1.03), while continuous PCS score had the highest increase in prevalence associated with lost workdays (APR = 1.03, 95% CI: 1.03, 1.03).

## Discussion

Findings suggest that physical and mental HRQOL were associated with all selected military readiness measures of interest. These results align with findings from the Millennium Cohort Family Study, a related study of military spouses, where physical and mental HRQOL had consistent direct effects on military spouse readiness (i.e., military satisfaction, lost workdays, health care utilization, military-related stress, and satisfaction) [59]. Additionally, when evaluated among just female service members of this study population, the same associations were found; lower physical and mental HRQOL scores were associated with decreased readiness as measured by obese BMI and more lost workdays [52]. While physical and mental HRQOL measures may not be the only predictors of readiness, they are predictors of poor readiness, even when accounting for health behaviors.

High physical HRQOL scores were associated with all indicators of military readiness except positive military satisfaction. Low physical HRQOL scores were associated with all measured predictors of readiness, not solely with physical indicators of poor readiness. Previous findings from the Millennium Cohort Study found that physical HRQOL was lower among service members injured during deployment [7], suggesting that service members with low physical HRQOL may have more medical health care needs, resulting in lost workdays or more time spent seeking health care.

Our analyses showed that having a high mental HRQOL score was associated with a non-obese BMI at follow-up, higher likelihood of remaining in service or separating for routine reasons, and positive military satisfaction. A higher mental HRQOL was not associated with other readiness indicators. Having a lower mental HRQOL score was negatively associated with all indicators of readiness. Poor mental health may inhibit service members’ engagement in health-promoting behaviors [60], increasing the risk of having obesity and related chronic conditions. Those with lower mental HRQOL scores were also more likely to have separated before completing their service term, consistent with prior research showing that service members diagnosed with or hospitalized for mental health reasons were more likely to leave service within 6 months of the incident [61]. The consistency of associations between mental HRQOL and all measures of readiness suggest that mental well-being is an important part of the comprehensive fitness requirements needed for service members to succeed in the military occupational environment.

While this study had notable strengths, including its longitudinal design, inclusion of service members from all service branches, use of military and electronic health records, and strong statistical power due to the large sample size, there are also limitations to note. Participants were required to complete two study surveys and remain on active duty during both study cycles, which limited the sample size and generalizability of study findings and may have introduced a healthy survivor bias. Second, while there is no “gold standard” measure of readiness, the measures used in this study may not fully encompass military readiness. However, the measures selected for this study were selected to reflect multiple physical and mental facets of military readiness [1,35,36]. Also, many survey measures are self-reported and may be susceptible to recall error or bias. The military has a strong culture of stoicism; these cultural biases, including a desire to appear strong and mask vulnerabilities, may result in underreporting of physical and mental health issues [62]. Also, it is important to note that while high utilization of non-preventive health care may be an indicator of lack of readiness, low utilization is more nuanced and indicative of multiple, potentially conflicting factors. For example, low utilizers may include service members who do not need non-preventive health care, but also may include those facing barriers to care such as lack of time or child care, or those concerned with potential stigma or career limitations due to care seeking [63]. This study includes many baseline health behaviors such as sleep, drinking, and mental health measures (e.g., baseline probable PTSD and depression), but does not include a comprehensive physical fitness measure such as a physical fitness assessment, which was unavailable for this study and may confound the association between HRQOL and readiness measures.

## Conclusion

This study examined HRQOL and health behaviors in relation to several measures of readiness in a large, contemporary study of service members. Findings suggest that HRQOL has the potential to be a useful tool to monitor readiness of service members. Routine assessment of HRQOL starting at baseline and with repeated measures during annual wellness exams in a clinical setting, for example, could be utilized to identify and monitor changes in service member readiness. The brevity of the VR-12 and the consistency of its association with readiness outcomes after adjustment for covariates suggests that it can be an efficient and practical tool in identifying service members who may be struggling with physical or mental readiness. Further research is needed to corroborate the utility of HRQOL as a screening tool to identify individuals with diminished military readiness and to identify actions that can improve HRQOL and subsequently, readiness.
